# Supplementary material for: TANGO6 regulates cell proliferation via COPI vesicle-mediated RPB2 nuclear entry
Source: Nat Commun. 2024 Mar 15;15:2371. doi: 10.1038/s41467-024-46720-y (PMC10943085; doi:10.1038/s41467-024-46720-y)
Supplement: Supplementary file 1 — Supplementary Information [file 41467_2024_46720_MOESM1_ESM.pdf]

Supplementary information for

TANGO6 regulates cell proliferation via COPI vesicle-mediated RPB2 nuclear entry

Zhi Feng[1†], Shengnan Liu[2†], Ming Su[1], Chunyu Song[2], Chenyu Lin[2], Fangying Zhao[2], Yang Li[1], Xianyan Zeng[3], Yong Zhu[3], Yu Hou[3], Chunguang Ren[3], Huan Zhang[3], Ping Yi[4], Yong Ji[5,6], Chao Wang[7], Hongtao Li[2], Ming Ma[2], Lingfei Luo[2*] and Li Li[1*]

†These authors contributed equally to this work.

*Corresponding author. E-mail: lili@cigit.ac.cn (L. Li), lluo@swu.edu.cn (L. Luo)

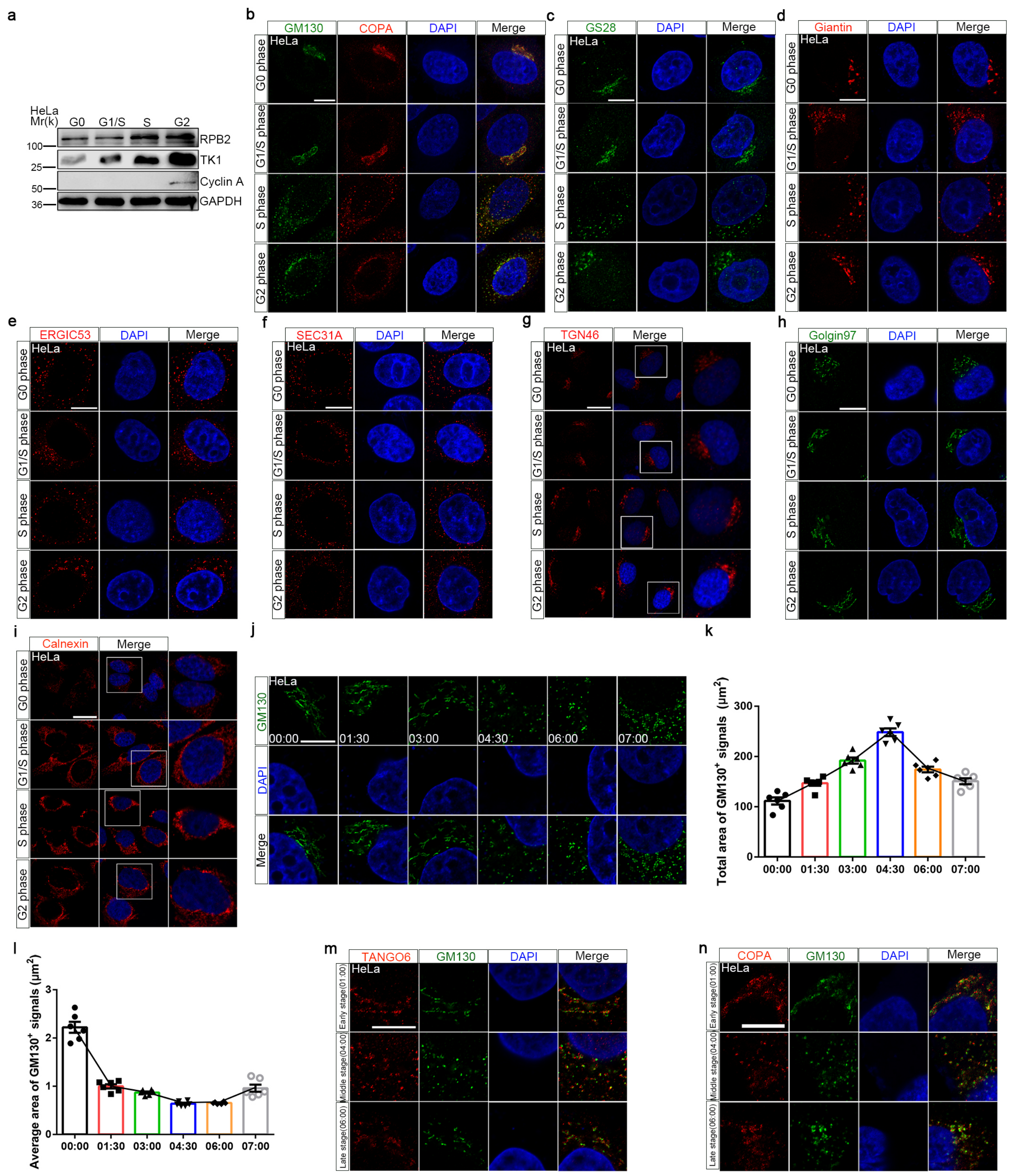

Supplementary Figure 1| The dynamics of Golgi and ER organelles in the interphase of cell cycle. a, Western blot analysis of RPB2, TK1 and Cyclin A levels in G0, G1/S, S and G2 phases. GAPDH is the internal standards. **b,c,d,e,f,g,h,i,** The immunofluorescent staining images of GM130 and COPA (**b**), GS28 (**c**), Giantin (**d**), ERGIC53 (**e**), Sec31A (**f**),TGN46 (**g**), Golgin97 (**h**), and Calnexin (**i**) in G0, G1/S, S and G2 phases. The right panels in (**g**) and (**i**) are the enlarged field of boxed cells. Scale bar, 10 μm. **j,** The immunofluorescent staining images of GM130 during S phase (00:00-07:00). Scale bar, 10 μm. **k,l,** The quantifications of total **(k)** and average **(l)** areas of GM130^{+}

signals (total areas: 00:00, 111.60μm^2 ± 7.08 μm^2; 01:30, 146.90μm^2 ± 5.13 μm^2; 03:00, 191.80 μm^2 ± 6.02 μm^2; 04:30, 248.30 μm^2 ± 7.54 μm^2; 06:00, 174.10 μm^2 ± 5.54 μm^2; 150.80 μm^2 ± 5.83 μm^2. n=6; average areas: 00:00, 2.22 μm^2 ± 0.11 μm^2; 01:30, 0.99 μm^2 ± 0.04 μm^2; 03:00, 0.87 μm^2 ± 0.02 μm^2; 04:30, 0.64 μm^2 ± 0.02 μm^2; 06:00, 0.65 μm^2 ± 0.01 μm^2; 0.96 μm^2 ± 0.07 μm^2. Mean ± s.e.m, n=6 cells). **m,n,** The immunofluorescent staining images of GM130 and TANGO6 (**m**) /COPA (**n**) in the early (01:00), middle (04:00) and late (06:00) stages of S phase. Scale bar, 10 μm. Source data are provided as a Source Data file.

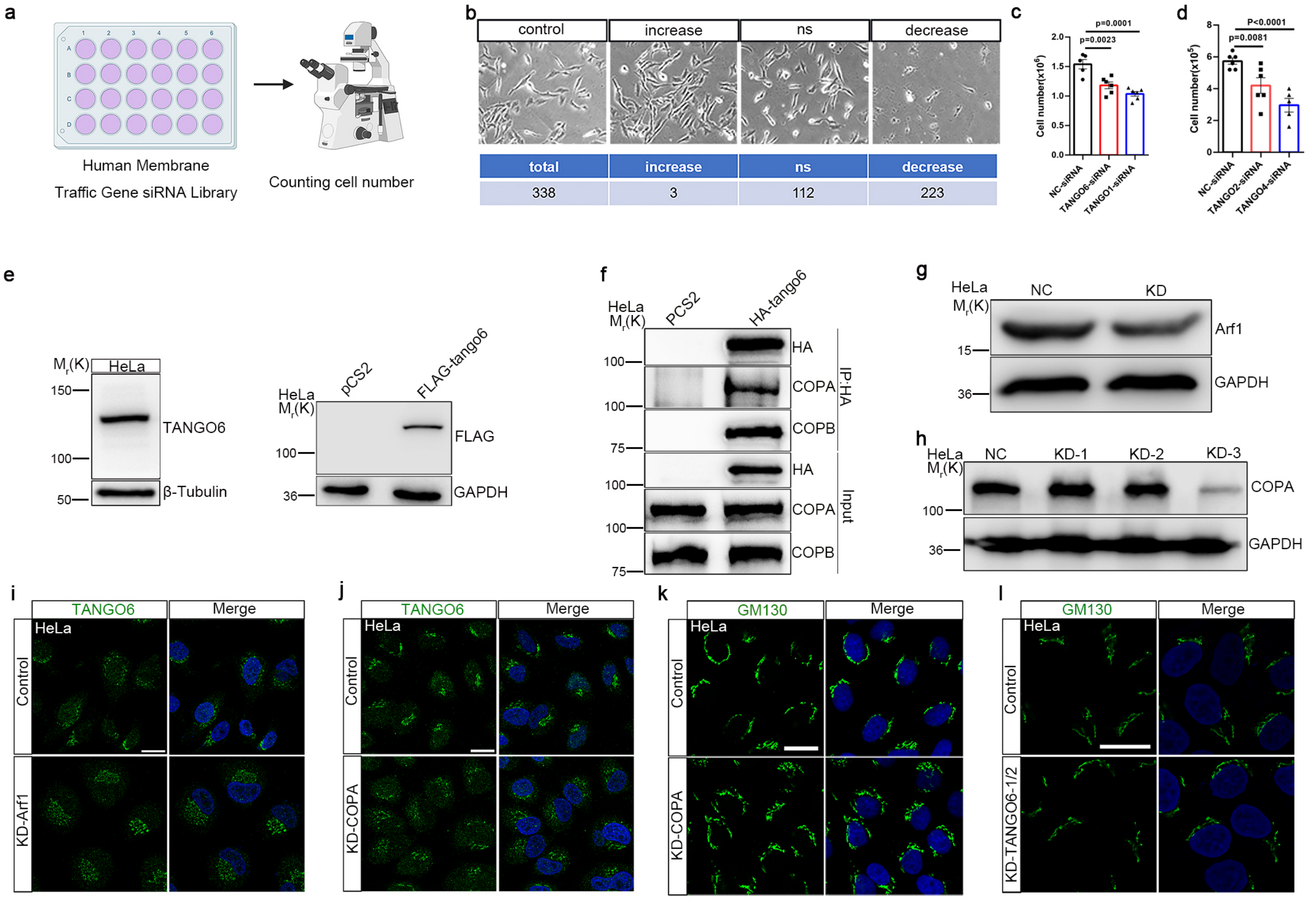

Supplementary Figure 2| The signatures of COPI and Golgi complex upon aberration of TANGO6 in the siRNA library screening. a, The schematic diagram of siRNA library screening process. **b,** Wide-field microscope images of different cell density (up) and the outcome summary (bottom). **c,d,** Statistical results of cell number after KD-TANGO1/6 (**c**) and KD-TANGO2/4 (**d**). (**c,** NC, 1.54 ± 0.08; KD-TANGO6, 1.18 ± 0.06; KD-TANGO1, 1.04 ± 0.04; **d**, NC, 5.73 ± 0.19; KD-TANGO2, 4.20 ± 0.49; KD-TANGO4, 2.96 ± 0.43.n=5/6. Each point in (**c,d**) denotes the number of cells in a visual field). **e,** Western blot analysis shows the protein molecular weight of endogenous (left) and FLAG-tagged (right) TANGO6. β-Tubulin and GAPDH are internal standards. **f,** Co-immunoprecipitation of TANGO6 with COPA and COPB. **g,h,** Western blot analysis of Arf1 (**g**) and COPA (**h**) after knocking down by corresponding siRNAs. GAPDH is internal standard. **i,j,** The immunofluorescent staining images of TANGO6 distribution after knocking down Arf1 (**i**) and COPA (**j**). **k,l,** $GM130^+$ signals distribution after knocking down COPA (**k**) and TANGO6 (**l**) respectively. Scale bar, 20 μm. Statistical significance for (**c,d**) were assessed using unpaired one-tailed Student's t-test. Mean ± s.e.m, ns, no significance. Source data are provided as a Source Data file.

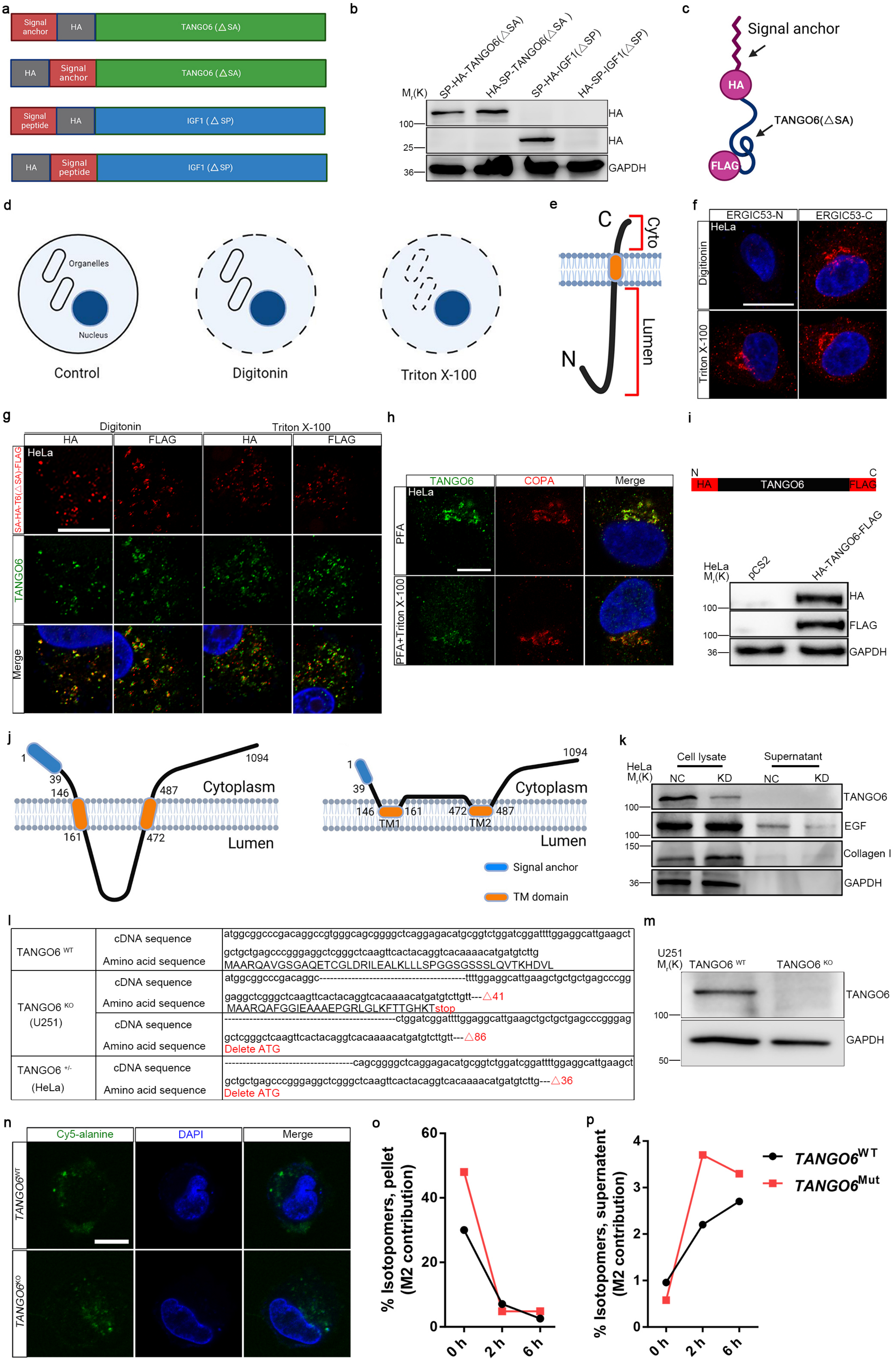
a
Signal anchor
HA
TANGO6 (△SA)
Signal peptide
IGF1 (△SP)
b
SP-HA-TANGO6(△SA)
HA-SP-TANGO6(△SA)
SP-HA-IGF1(△SP)
HA-SP-IGF1(△SP)
Mr(K)
100
25
36
HA
GAPDH
c
Signal anchor
HA
TANGO6(△SA)
FLAG
d
Organelles
Nucleus
Control
Digitonin
Triton X-100
e
C
Cyto
N
Lumen
f
ERGIC53-N
ERGIC53-C
HeLa
Digitonin
Triton X-100
g
Digitonin
Triton X-100
HA
FLAG
HeLa
SA-HA-T6(△SA)-FLAG
TANGO6
Merge
h
TANGO6
COPA
Merge
HeLa
PFA
PFA+Triton X-100
i
N
C
HA
TANGO6
FLAG
HeLa
Mr(K)
pCS2
HA-TANGO6-FLAG
100
36
HA
FLAG
GAPDH
j
1
39
146
161
472
487
1094
Cytoplasm
Lumen
TM1
TM2
Signal anchor
TM domain
k
HeLa
Mr(K)
Cell lysate
Supernatant
NC
KD
100
150
36
TANGO6
EGF
Collagen I
GAPDH
l
TANGO6 WT
cDNA sequence
Amino acid sequence
atggcggcccgacaggccgtgggcagcggggctcaggagacatgcggtctggatcggattttggaggcattgaagct
gctgctgagcccgggaggctcgggctcaagttcactacaggtcacaaaacatgatgtcttg
MAARQAVGSGAQETCGLDRILEALKLLLSPGGSGSSSLQVTKHDVL
TANGO6 KO
(U251)
atggcggcccgacaggc--ttttggaggcattgaagctgctgctgagcccgg
gaggctcgggctcaagttcactacaggtcacaaaacatgatgtcttgtt---△41
MAARQAFGGIEAAAEPGRLGLKFTTGHKTstop
--ctggatcggattttggaggcattgaagctgctgctgagcccgggag
gctcgggctcaagttcactacaggtcacaaaacatgatgtcttgtt---△86
Delete ATG
TANGO6 +/-
(HeLa)
-----------------------------------cagcggggctcaggagacatgcggtctggatcggattttggaggcattgaagct
gctgctgagcccgggaggctcgggctcaagttcactacaggtcacaaaacatgatgtcttg---△36
Delete ATG
m
U251
Mr(K)
TANGO6 WT
TANGO6 KO
100
50
TANGO6
GAPDH
n
Cy5-alanine
DAPI
Merge
TANGO6WT
TANGO6KO
o
% Isotopomers, pellet (M2 contribution)
60
40
20
0
0 h
2 h
6 h
p
% Isotopomers, supernatent (M2 contribution)
4
3
2
1
0
0 h
2 h
6 h
TANGO6WT
TANGO6Mut

Supplementary Figure 3| Characterization of signal anchor and the topology diagram of TANGO6. a, The diagram of four constructs (signal anchor/peptide-HA-TANGO6$^{\triangle SA}$/IGF1$^{\triangle SP}$ and HA-signal peptide/anchor-TANGO6$^{\triangle SA}$/IGF1$^{\triangle SP}$). SA/P, signal anchor/peptide. **b,** Western blot analysis of HA after delivering SA-HA/HA-SA-TANGO6 (△SA) and SP-HA/HA-SP-IGF1(△SP) plasmids. GAPDH is internal standard. **c,** The schematic diagram of SA-HA-TANGO6 (△SA)-FLAG construct. **d,** The schematic diagram of membrane permeation after digitonin and Triton X-100 treatment. **e,f,** The diagram of ERGIC53 topology (**e**) and immunofluorescent staining images of ERGIC53 antibodies (**f**) (antagonize N or C terminals) in digitonin or Triton X-100 treated cells. Scale bar, 10 μm. **g,** The immunofluorescent staining images of HA-SA-TANGO6 (△SA)-FLAG and TANGO6 distribution after digitonin or Triton X-100 treatment. Scale bar, 10 μm. **h,** The immunofluorescent staining images of TANGO6 and COPA in PFA or PFA plus Triton X-100 treated cells. Scale bar, 10 μm. **i,** Validation of HA-TANGO6-FLAG expression by western blot analysis. GAPDH is the internal standard. **j,** The putative topology models of TANGO6. The signal anchor is marked by blue color and transmembrane (TM) domain is marked by orange color. **k,** The western blot analysis of EGF and collagen I in the cell lysates or supernatants after knocking downTANGO6. GAPDH is the internal standard. **l,** cDNA and amino acid sequence of *TANGO6*^{KO} and *TANGO6*$^{+/-}$. For *TANGO6*^{KO}, one allele deletes 41 bp and leads to the early termination of translation and the other allele deletes 86 bp (includes ATG). For *TANGO6*$^{+/-}$, one allele deletes 36 bp (includes ATG) and the other allele is normal. **m,** Western blot analysis of TANGO6 in *TANGO6*^{WT} and *TANGO6*^{KO} cells. GAPDH is used as internal standard. **n,** The immunofluorescent staining images of Cy5-alanine in *TANGO6*^{WT} and*TANGO6*^{KO} cells. Scale bar, 10 μm. **o,p,** The statistical graph of[2,3-$^{13}C_2$]alanine (M2) relative abundance in cell pellet (**o**) or medium supernatant (**p**) at different time points. Source data are provided as a Source Data file.

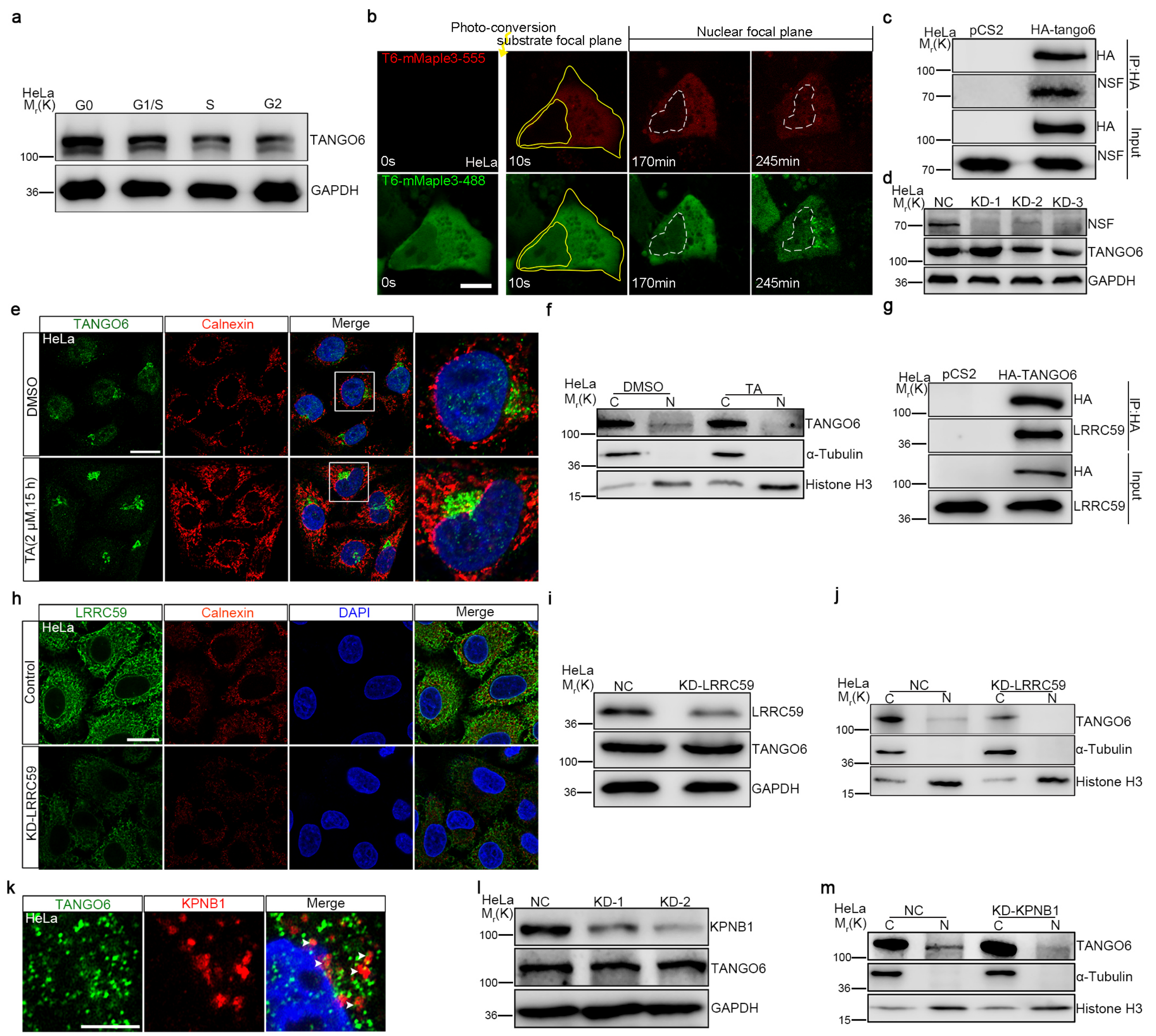

Supplementary Figure 4| TANGO6 nuclear entry depends on NSF, LRRC59 and KPNB1. a, Western blot analysis of TANGO6 in different interphases (G0, G1/S, S and G2) of cell cycle. GAPDH is internal standard. **b,** Time-lapse imaging of the photo-converted TANGO6-mMaple3 in HeLa cells. Photo-converting laser is focused on the bottom of the cell (the photo-converting area was indicated by yellow line). The TANGO6-mMaple3^{+} signals were monitored in the nuclear focal plane (white dashed line). Scale bar, 10 μm. **c,** Co-immunoprecipitation of TANGO6 with NSF. **d,** Western blot analysis of NSF and TANGO6 after knocking down NSF by siRNA. GAPDH is internal standard. **e,** The immunofluorescent staining images of TANGO6 and Calnexin after treating with Thapsigargin (TA). The right panels are amplified images of boxed region in the left. The white arrowheads indicate nuclear TANGO6^{+} signals. Scale bar, 20 μm. **f,** Western blot analysis of TANGO6 distribution in cytoplasm and nucleus after TA treatment. β-Tubulin and Histone H3 are

internal standards. C, cytoplasm; N, nucleus. **g,** Co-immunoprecipitation of TANGO6 and LRRC59. **h,** Immunofluorescent staining images of LRRC59 and Calnexin distribution after transfecting LRRC59 siRNA. Scale bar, 20 μm. **i,j,** Western blot analysis of total TANGO6 (**i**) or cytoplasmic and nuclear TANGO6 distribution (**j**) after knocking down LRRC59. GAPDH is internal standard. β-Tubulin and Histone H3 are internal standards. **k,** The immunofluorescent staining images of TANGO6 and KPNB1. The white arrowheads indicate merged signals. Scale bar, 10 μm. **l,m,** Western blot analysis of total TANGO6 (**l**) or cytoplasmic and nuclear TANGO6 distribution (**m**) after knocking down KPNB1. GAPDH is internal standard. β-Tubulin and Histone H3 are internal standards. Source data are provided as a Source Data file.

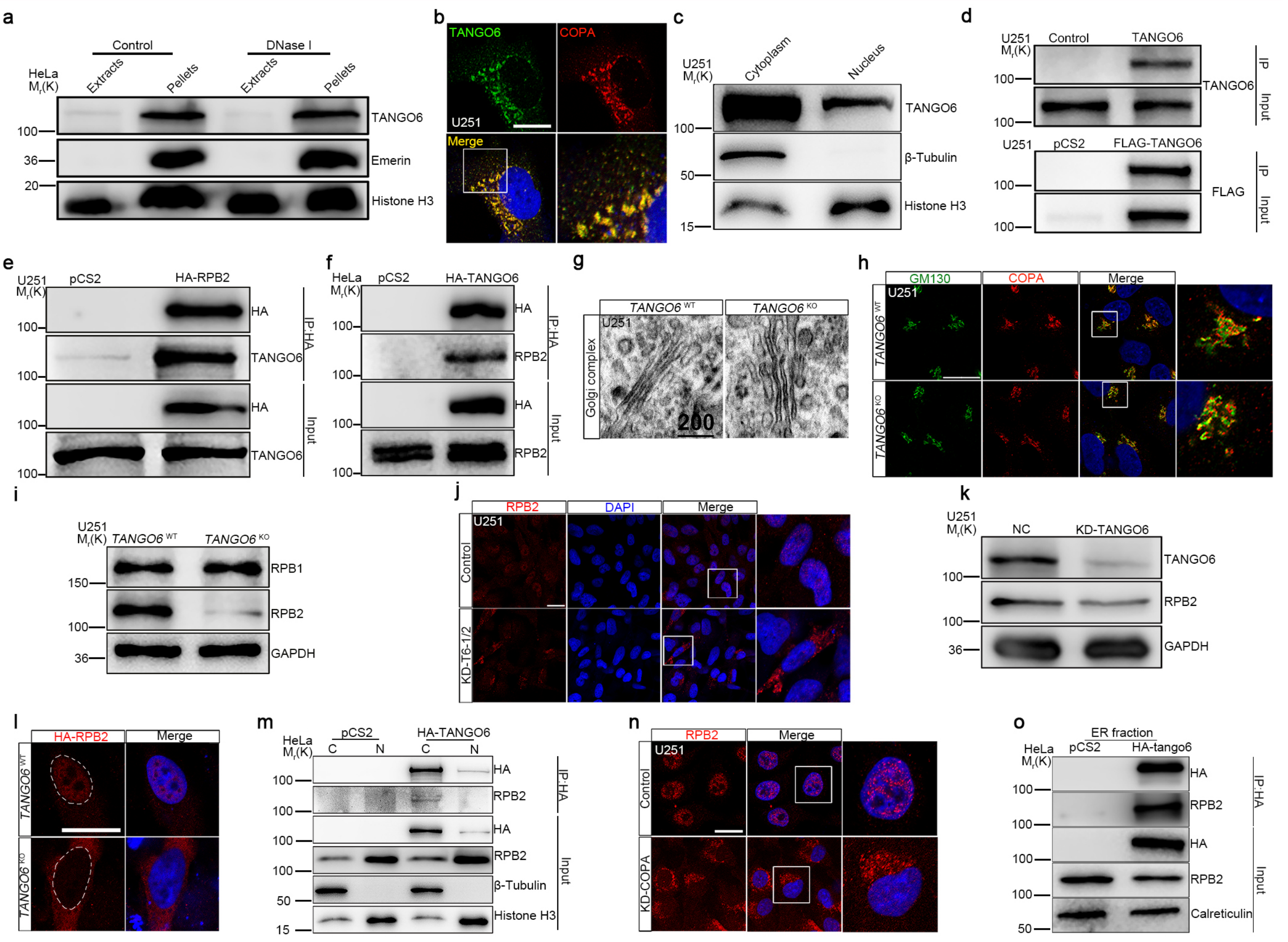

Supplementary Figure 5| Validation of TANGO6 interacting proteins and *TANGO6*^{KO} construction. a, Western blot analysis of TANGO6 activity in nuclear soluble extract and pellet after DNase I treatment. Emerin and Histone H3 are used as internal standards. **b,** Immunofluorescent staining images of TANGO6 and COPA distribution in the U251 cells. The bottom-right corner is the enlarged field of boxed cells. Scale bar, 10 µm. **c,** The cytoplasmic and nuclear TANGO6 distribution in U251 cells by western blot analysis. **d,** Pulling down endogenous TANGO6 (top) and exogenous FLAG-TANGO6 (bottom) by immunoprecipitation. **e,f,** Validation of RPB2 and TANGO6 interaction by co-immunoprecipitation assay in the U251 cells (**e**) and HeLa cells (**f**). **g,** Golgi apparatus structure in *TANGO6*^{WT} and *TANGO6*^{KO} cells by transmission electron microscope. Scale bar, 200 nm. **h,** The immunofluorescent staining images of GM130 and COPA in *TANGO6*^{WT} and *TANGO6*^{KO} cells. The right panel is the enlarged field of boxed cells. Scale bar, 20 µm. **i,** Western blot analysis of RPB1 and RPB2 in *TANGO6*^{KO} cells. GAPDH is used as internal standard. **j,** The immunofluorescent staining images of RPB2 distribution after knocking down TANGO6 in the U251 cells. The right panel is the enlarged field of boxed cells. Scale bar, 20 µm. **k,** Western blot analysis

of TANGO6 and RPB2 after knocking down TANGO6 in the U251 cells. GAPDH is used as internal standard. **l,** The immunofluorescent staining images of HA-RPB2 in *TANGO6* ^{WT} and *TANGO6* ^{KO} cells. The dashed line indicates cell nucleus region. Scale bar, 10 μm. **m,** Co-immunoprecipitation of HA-TANGO6 with RPB2 in cell cytoplasm (C) and nucleus (N). β-Tubulin and Histone H3 are used as internal standards of cytoplasmic and nuclear proteins respectively. **n,** The immunofluorescent staining images of RPB2 in COPA knock-down (KD) cells. The right panels are the enlarged field of boxed cells. Scale bar, 20 μm. **o,** Co-immunoprecipitation of HA-TANGO6 with RPB2 in ER fraction. Calreticulin is used as internal standard. Source data are provided as a Source Data file.

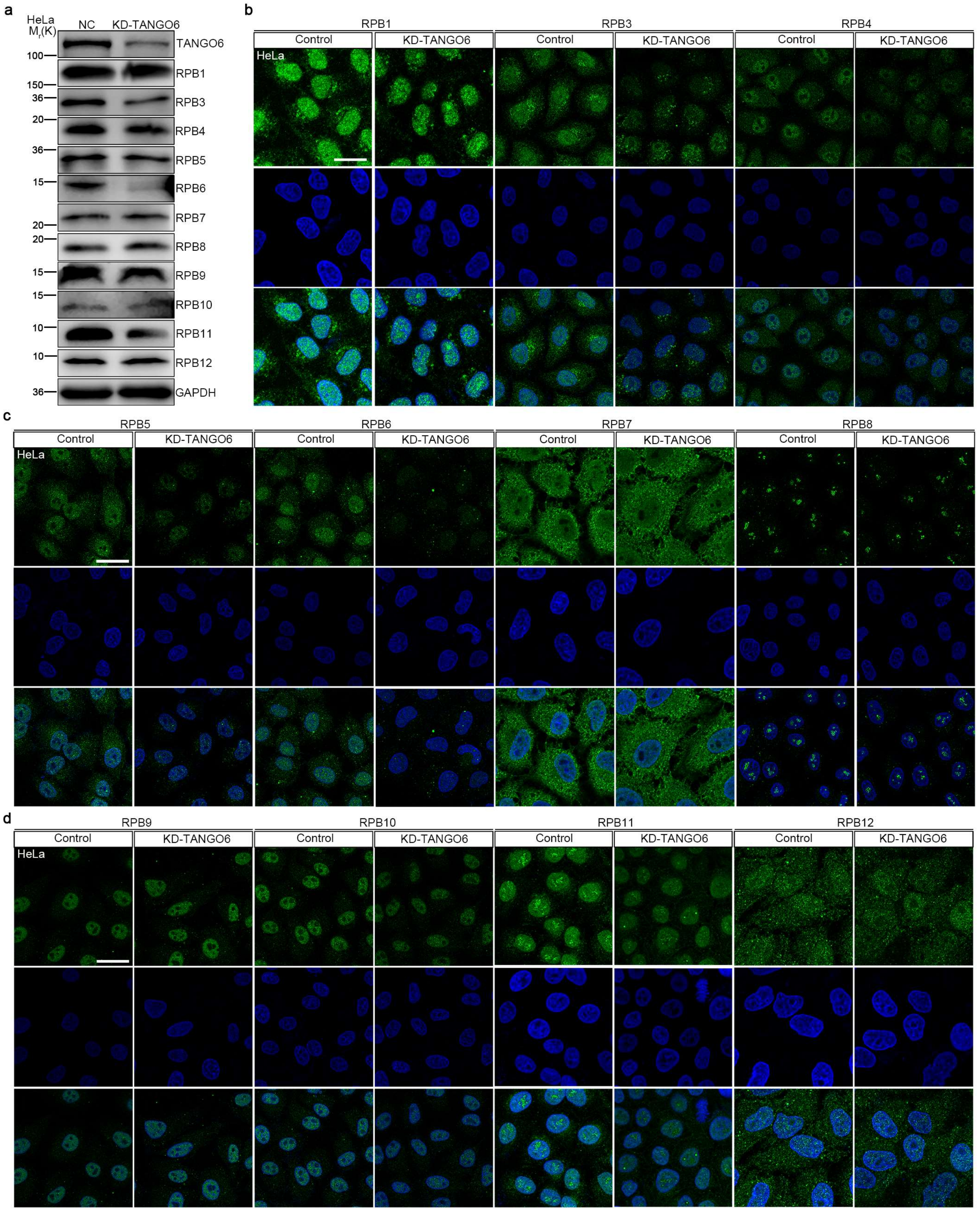

Supplementary Figure 6| The levels and distributions of RNA polymerase II subunits (excepting RPB2) after knocking down TANGO6. a, Western blot analysis of RPB1-12 activity after knocking down TANGO6 in HeLa cells. GAPDH is the internal standard. **b,c,d,** The immunofluorescent staining images of RPB1/3/4 (**b**), RPB5-8 (**c**) and RPB9-12 (**d**) distributions after

knocking down TANGO6 in HeLa cells. Scale bar, 20 μm. Source data are provided as a Source Data file.

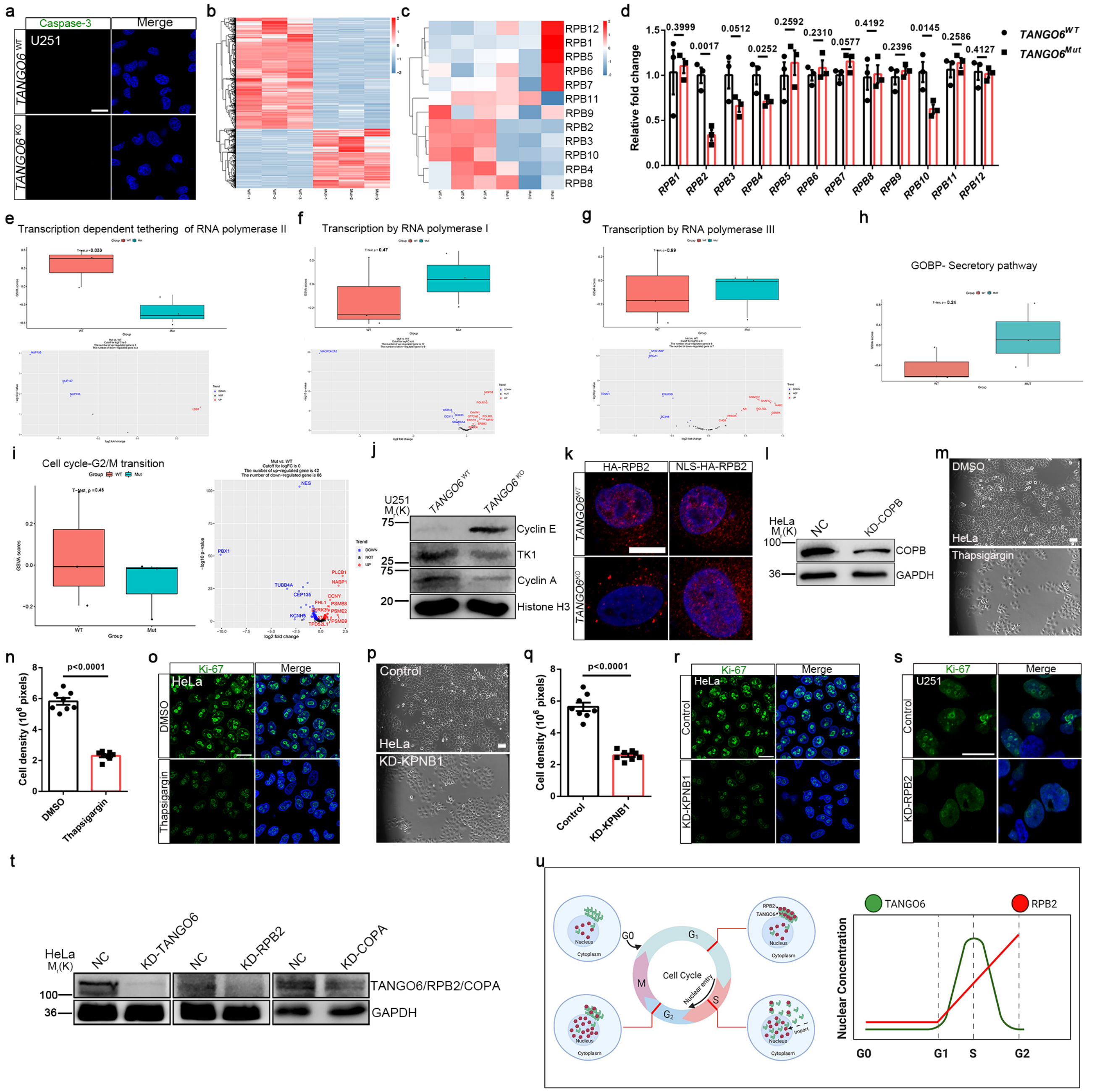

Supplementary Figure 7| RNA-seq of TANGO6 deficiency and the role of ER, KPNB1 and RPB2 in cell proliferation. a, Immunofluorescent staining images of active Caspase 3 in *TANGO6* ^{WT} and *TANGO6* ^{KO} cells. Scale bar, 20 μm. **b,c,** The heatmaps of differentially expressed genes (DEGs) clusters (**b**) and each subunit of RNA polymerase II (**c**) in *TANGO6* ^{WT} and *TANGO6* ^{KO} cells. **d,** qRT-PCR results of each subunit of RNA polymerase II in *TANGO6* ^{WT} and *TANGO6* ^{KO} cells. (n=3 independent experiments). **e,f,g,** GSVA analysis of transcriptional process by RNA polymerase II (**e**), I (**f**) and III (**g**) between *TANGO6* ^{WT} and *TANGO6* ^{KO} cells. The top panel is the GSVA scores and the down pane is related genes enriched in these processes. **h,** GSVA analysis of secretory pathway between *TANGO6* ^{WT} and *TANGO6* ^{KO} cells. **i,** GSVA analysis of G2/M transition process in cell cycle progression. The left panel is the GSVA scores and the right panel is the related genes

enriched in this process. **j,** Western blot analysis of Cyclin E, Thymidine Kinase 1 (TK 1) and Cyclin A in the *TANGO6* ^{WT} and *TANGO6* ^{KO} cells. Histone H3 is used as internal standard. **k,** The immunofluorescent staining images of HA-RPB2 or NLS-HA-RPB2 in *TANGO6* ^{WT} and *TANGO6* ^{KO} cells. Scale bar, 10 μm. **l,** Western blot analysis of COPB after knocking down COPB. GAPDH is used as internal standard. **m,n,o,p,q,r,** The wide field microscope images and corresponding cell density statistics (**m,n**), the immunofluorescent staining images of Ki-67 (**o**) after Thapsigargin treatment or knocking down KPNB1 by siRNA (**p,q,r**). (n=8. Each point in (**n,q**) denotes the cell density in a visual field). Scale bar, 20 μm**. s,** The immunofluorescent staining images of Ki-67 after knocking down RPB2 in the U251 cells. Scale bar, 20 μm. **t,** Western blot analysis of TANGO6, RPB2 and COPA after transfecting corresponding siRNA. GAPDH is internal standard. **u,** The diagram of TANGO6 and RPB2 distribution (left) and nuclear concentration (right) during interphase of cell cycle (G0, G1, S, G2) progression. Source data are provided as a Source Data file.

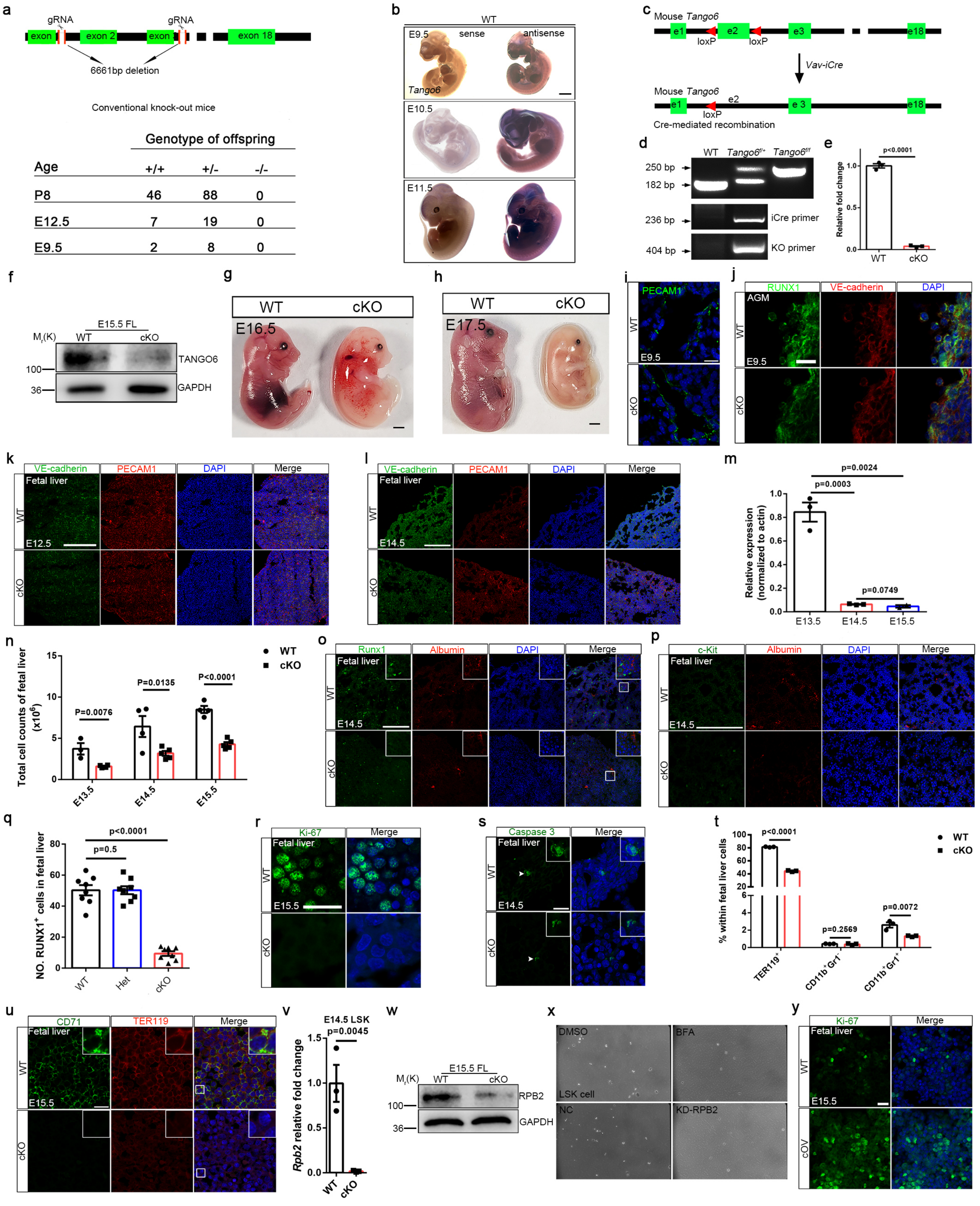

Supplementary Figure 8| Expression pattern and generation of *Tango6* knock-out mice. a, A schematic representation of conventional knock-out mice design. The whole exon 2 and exon 3 are deleted. Green rectangles indicate the exons and black rectangles indicate the introns (top). Genotype identification of the offspring from the intercross of *Tango6* conventional knock-out mice (bottom). **b**, (WISH) of *Tango6* at different stages (E9.5, E10.5, E11.5) of mice. Scale bar, 100 µm. **c**, A

schematic representation of *Tango6* conditional knock-out mice (cKO) generation. e, exon. **d,** Genotyping of PCR analysis. wild type locus, 182 bp; the floxed locus, 250 bp; the targeted copy, 236 bp; the excised copy, 404 bp. **e,** qRT-PCR of *Tango6* in the LSK ($Lin^{-}Sca\text{-}1^{+}c\text{-}Kit^{+}$) cells of E15.5 fetal liver (WT, 1.00 ± 0.03; cKO, 0.04 ± 0.01. n=3. Each dot represents an independent experiment.). **f,** Western blot analysis of TANGO6 in the fetal liver of WT and cKO mice. **g,h,** Photographic images of WT and cKO embryos at E16.5 (**g**) and E17.5 (**h**). Scale bar, 2 mm. **i,j,** The immunofluorescent staining images of PECAM1 (**i**) and VE-cadherin/RUNX1 (**j**) in AGM regions in E9.5 mice embryos. Scale bar, 20 μm. **k,l,** The immunofluorescent staining images of VE-cadherin and PECAM1 in E12.5 (**k**) and E14.5 (**l**) mice embryos. Scale bar, 20 μm. **m,** qRT-PCR of *Tango6* at different stages (E13.5, 0.85 ± 0.08; E14.5, 0.06 ± 0.00; E15.5, 0.05 ± 0.01. n=2/3 independent experiments. Each dot represents an independent experiment.). **n,** Statistical graph of total fetal liver cell counts from E13.5 to E15.5. (E13.5, WT, 3.73 ± 0.69; cKO, 1.59 ± 0.08; E14.5, WT, 6.43 ± 1.27; cKO, 3.18 ± 0.29; E15.5, WT, 8.49 ± 0.43; cKO, 4.29 ± 0.26. n=3/4/5 mice). **o,p,** The double staining images of Albumin with Runx1 (**o**) or c-Kit (**p**). Scale bar, 200 μm. **q,** The number of $Runx1^{+}$cells in the E15.5 fetal livers (WT, 50.25 ± 3.32; Het (heterozygous, Vav-iCre/$Tango6^{f/+}$), 50.25 ± 2.60; cKO (homozygous, Vav-iCre/$Tango6^{f/f}$) 9.38 ± 1.48. n=8 visual field in each group). Each point in (q) denotes the number of Runx1+ cells in a visual field.). **r,s,** The immunofluorescent staining images of Ki-67 (**r**) and active-Caspase 3 (**s**) in WT and cKO mice at E15.5 stage. Scale bar, 20 μm. **t,** Representative flow cytometric profile of myeloid cells and erythrocytes of E15.5 fetal livers (TER119+, WT, 81.37% ± 0.30%; cKO, 44.37% ± 0.62%; CD11b+Gr1-, WT, 0.43% ± 0.02%; cKO, 0.37% ± 0.07%; CD11b+Gr1+, WT, 2.60% ± 0.31%; cKO, 1.32%± 0.05%. n=3 mice in each group). **u,** The immunofluorescent staining images of CD71 and TER119 in the fetal livers of E15.5 mice embryos. Scale bar, 20 μm. **v,w,** qPCR (**v**) and western blot analysis (**w**) of RPB2 levels in WT and cKO mice at E15.5. (v, WT, 0.99 ± 0.21; cKO, 0.02± 0.004. n=3 independent experiments. Each dot represents an independent experiment.). **x,** The wild-field microscope images of *in vitro* cultured HSPCs after BFA treatment or knocking down RPB2. **y,** The immunofluorescent staining images of Ki-67 in WT and cOV (conditional overexpression) mice. Scale bar, 20 μm. Statistical significance

for (**e,m,n,q,t,v**) were assessed using unpaired one-tailed Student's t-test. Mean ± s.e.m. Source data are provided as a Source Data file.

Supplementary Table 1 List of primers used in this study

Name	Sequence (5' to 3')
Cyclin B -qPCR Forward	AAGGTGCCTGTGTGTGAAC
Cyclin B -qPCR Reverse	GTCAGCCCCATCATCTGCG
Cyclin D -qPCR Forward	ACACCGACAACTCTGTGAAGC
Cyclin D -qPCR Reverse	GCCAGGTTCCACTTCAGCTTA
PCNA -qPCR Forward	CCACATTGGAGATGCTGTTG
PCNA -qPCR Reverse	CAGTGGAGTGGCTTTTGTGA
Ki-67 -qPCR Forward	CTCCACGAACCTCAAAGA
Ki-67- qPCR Reverse	TGTGGATTCCTTCACACCTT
mouse-Tango6 -qPCR Forward	CAGGATGTTGTACGTCTGGA
mouse-Tango6 -qPCR Reverse	ATCCAAGATGTCTCGCAAGGC
β-actin -qPCR Forward	GAGACCTTCAACACCCCAGC
β-actin -qPCR Reverse	ATGTCACGCACGATTTCCC
Tango6 conventional knockout genotype of mut mice Forward	GTCAGCGGTGCATTTTCTTCTTTATG
Tango6 conventional knockout genotype of mut mice Reverse	CAGCAGGACCACAGATGCAAAAC
Tango6 conventional knockout genotype of WT mice Forward	GTCAGCGGTGCATTTTCTTCTTTATG
Tango6 conventional knockout genotype of WT mice Reverse	GTATAAATACCAGAGCCCCAGG
loxp of Tango6 conditional knockout mice Forward	CTTCCTGGTGTACAGTTTGGGTA
loxp of Tango6 conditional knockout mice Reverse	CAAACTGAGCTCCTTCCACAGAA
KO of Tango6 conditional knockout mice Forward	CTTCCTGGTGTACAGTTTGGGTA
KO of Tango6 conditional knockout mice Reverse	GCAGGGAGAATAGAAGTTCCAAGAA
IPC of Vav-iCre Forward	CTAGGCCACAGAATTGAAAGATCT
IPC of Vav-iCre Reverse	GTAGGTGGAAATTCTAGCATCATCC
TG of Vav-iCre Forward	AGATGCCAGGACATCAGGAACCTG
TG of Vav-iCre Reverse	ATCAGCCACACCAGACACAGAGATC
Tango6 conditional overexpression mice Forward-1	CACTTGCTCTCCCAAAGTCGCTC
Tango6 conditional overexpression mice Reverse-1	ATACTCCGAGGCGGATCACAA
Tango6 conditional overexpression mice Forward-2	GGACTCAGCCAGAAAGCAACAG
Tango6 conditional overexpression mice Reverse-2	TTCAGGGTCAGCTTGCCGTAG
Tango6 conditional overexpression mice Forward-3	AGATCTGCAAGCTAATTCCTGC
Tango6 conditional overexpression mice Reverse-3	GTAGTGAACTTGAGCCTGATCCT
POLR2A-qPCR Forward	GGGTGGCATCAAATACCCAGA

POLR2A-qPCR Reverse	AGACACAGCGCAAAACTTTCA
POLR2B-qPCR Forward	GCGGATGAGGATATGCAATATGA
POLR2B-qPCR Reverse	ACCAAGCCTTTCTCGTCAAAA
POLR2C-qPCR Forward	CTCACTGACGAGAATGTCAAGTT
POLR2C-qPCR Reverse	TGAAGACCCTCCGAATCGAAT
POLR2D-qPCR Forward	CAGCCCGTTTCAGTCGTTTC
POLR2D-qPCR Reverse	CCAAACAGGCCAACTCAAACTT
POLR2E-qPCR Forward	TGGAAAATCCGCAAGACCATC
POLR2E-qPCR Reverse	CGCTGGCAGTACACCTTGAT
POLR2F-qPCR Forward	ATGTCAGACAACGAGGACAATTT
POLR2F-qPCR Reverse	TTCGGCATTCTCCAAGTCATC
POLR2G-qPCR Forward	ATCTCCCTAGAGCACGAAATCC
POLR2G-qPCR Reverse	ACAAAGCCATACTTCCCTGTG
POLR2H-qPCR Forward	GACAAGTTCCGGTTGGTCATAG
POLR2H-qPCR Reverse	AGTGGGGTTGTATTCACCATCA
POLR2I-qPCR Forward	GGCTTCGTGGGTATTCGCTT
POLR2I-qPCR Reverse	CTCCTGCTGGTAATCACAGTTC
POLR2J-qPCR Forward	CAGGAAGCCTTTACCAACGCCA
POLR2J-qPCR Reverse	CACAGGTAGGAACGGGGCTCA
POLR2K-qPCR Forward	GGAGAGTGTCACACAGAAAATGA
POLR2K-qPCR Reverse	TCGAGCATCAAAAACGACCAAT
POLR2L-qPCR Forward	TCACTTGTGGCAAGATCGTCG
POLR2L-qPCR Reverse	GGGTGCATAATTGAGCAGCTTC

Supplementary Table 2 List of antibodies used in this study

Name	Company	Cat#	Dilution(IF/WB)
Goat polyclonal anti-GFP	Abcam	ab6658	1:400; 1:2000
Goat polyclonal anti-Calnexin	Abcam	ab219644	1:400; 1:1000
Mouse monoclonal anti-beta-Tubulin	Invitrogen	MA5-11732	1:400; 1:2000
Goat anti-Mouse IgG (H+L) Secondary Antibody, HRP	Invitrogen	31430	1:3000
Goat anti-Rabbit IgG (H+L) Secondary Antibody,HRP	Invitrogen	32260	1:3000
Rabbit polyclonal anti-TMCO7 (TANGO6)	Invitrogen	PA5-59510	1:400; 1:1000
Alexa 488-Donkey anti-Goat IgG (H+L)	Invitrogen	A32814	1:800
Alexa 555-Donkey anti-Mouse IgG (H+L)	Invitrogen	A31570R	1:800
Alcxa 647-Donkey anti-Rabbit IgG (H+L)	Invitrogen	A31573	1:800
Mouse monoclonal anti-Lamin B1	Proteintech	66095-1-1g	1:400
Mouse monoclonal anti-PCNA	Proteintech	60097-1-1g	1:400; 1:1000
Mouse monoclonal anti-GAPDH	Proteintech	60004-1-Ig	1:2000
Rabbit Recombinant monoclonal anti-Histone 3	Bimake	A5885	1:2000
Mouse Recombinant monoclonal anti-HA	Bimake	A5969	1:1000
Rabbit Recombinant monoclonal anti-TGN46	Bimake	A5460	1:400; 1:1000
Rabbit Recombinant monoclonal anti-Calreticulin	Bimake	A5231	1:1000
Mouse Monoclonal anti-FLAG	Sigma-Aldrich	F1804	1:1000
Mouse monoclonal anti-COPA	Santa Cruz	SC-398099	1:200;1:500
Mouse monoclonal anti-COPB	Santa Cruz	SC-393615	1:200;1:500
Mouse monoclonal I anti-COPD	Santa Cruz	SC-515549	1:200;1:500
Mouse monoclonal anti-COPE	Santa Cruz	SC-133194	1:200;1:500
Mouse monoclonal anti-COPG	Santa Cruz	SC-393977	1:200;1:500
Mouse monoclonal I anti-COPZI	Santa Cruz	SC-398081	1:200;1:500
Mouse monoclonal anti-KPNB1	Santa Cruz	SC-137016	1:200;1:500
Mouse monoclonal: l anti-ERGIC53(F-3)	Santa Cruz	SC-398777	1:200;1:500
Mouse monoclonal anti-ERGIC53(B-4)	Santa Cruz	SC-398893	1:200;1:500
Mouse monoclonal anti-ERGIC53(C-6)	Santa Cruz	SC-365158	1:200;1:500
Mouse monoclonal anti-Sec3lA	Santa Cruz	SC-376587	1:200;1:500
Mouse monoclonal anti-Calnexin	Santa Cruz	SC-70481	1:100;1:400
Rabbit polyclonal anti-PH3	Santa Cruz	SC-8656	1:200;1:400
Mouse monoclonal anti-POLR2A (RPB1)	Santa Cruz	SC-55492	1:200; 1:1000

Mouse monoclonal anti-POLR2B (RPB2)	Santa Cruz	SC-166803	1:200;1:500
Mouse monoclonal anti-CDKI	Santa Cruz	SC-53219	1:200; 1:1000
Rabbit monoclonal anti-GM130	Cell Signaling Technology	124805	1:400; 1:1000
Rabbit monoclonal Ki-67(D3B5)	Cell Signaling Technology	9129T	1:400; 1:1000
Mouse monoclonal anti-GM130	BD Bioscience	610822	1:400; 1:1000
BD PharmingenTM Purified Rabbit Anti- Active Caspase-3	BD Bioscience	559565	1:400; 1:1000
Rabbit Polyclonal anti-Emerin	Proteintech	10351-1-AP	1:400; 1:1000
Mouse monoclonal anti-DsRed	Santa Cruz	SC-390909	1:200;1:500
Mouse monoclonal anti-Thymidine kinase 1	Santa Cruz	SC-377211	1:200;1:500
Mouse monoclonal anti-Cyclin E	Santa Cruz	SC-377100	1:200;1:500
Mouse monoclonal anti-Cyclin A	Santa Cruz	SC-271682	1:200;1:500
FITC anti-mouse Lineage Cocktail with Isotype Ctrl	Biolegend	133301	2 µL/105 cells
Rabbit monoclonal PE/Cyanine anti-mouse Ly-6A/E (Sca-1)	Biolegend	108113	0.2 µL/105 cells
Rabbit monoclonal APC anti-mouse CD117 (c-Kit)	Biolegend	105811	0.5 µL/105 cells
Rabbit monoclonal APC/Cyanine 7 anti-mouse TER-119/Erythroi	Biolegend	116223	1.25 µL/105 cells
Rabbit monoclonal APC anti-mouse Ly-6G/Ly-6C(Gr-1)	Biolegend	108411	1.25 µL/105 cells
Rabbit monoclonal FITC anti-mouse/human CD11b	Biolegend	101205	0.5 µL/105 cells
Alexa Fluor⑧ 700 anti-mouse Ki-67 Antibody	Biolegend	652419	0.03 µL/105 cells
Rabbit anti-Goat IgG (H+L) Secondary Antibody, HRP	Pierce	31402	1:200; 1:2000
abberior STAR RED	abberior	STRED	1:200
abberior STAR 580	abberior	ST580	1:200
Rabbit Polyclonal anti-Giantin	ABclonal	A22233	1:200
Rabbit monoclonal anti-VE Cadherin	ABclonal	A22659	1:200
Rabbit monoclonal anti-PECAM1	ABclonal	A19014	1:200
PE anti-mouse CD150 (SLAM) Antibody	Biolegend	162605	0.125 µL/105 cells
APC/Cyanine7 anti-mouse CD48 Antibody	Biolegend	103431	0.125 µL/105 cells
CLTC Monoclonal antibody	Proteintech	66487-1-Ig	1:400
POLR2I Polyclonal antibody	Proteintech	17270-1-AP	1:400
POLR2F Polyclonal antibody	Proteintech	15334-1-AP	1:200

POLR2C Polyclonal antibody	Proteintech	13428-1-AP	1:400
POLR2H Polyclonal antibody	Proteintech	15086-1-AP	1:400
POLR2J Polyclonal antibody	Proteintech	16403-1-AP	1:400
POLR2D Polyclonal antibody	Proteintech	16093-1-AP	1:400
POLR2L Polyclonal antibody	Proteintech	15779-1-AP	1:400
Anti-POLR2G Polyclonal Antibody	Solarbio	K004815P	1:200
Rabbit Polyclonal anti-POLR2K	GeneTex	GTX132871	1:400
GS28 Polyclonal antibody	Proteintech	16106-1-AP	1:400
golgin 97 Polyclonal antibody	Proteintech	12640-1-AP	1:400
LRRC59 Polyclonal antibody	Proteintech	27208-1-AP	1:400
CD31 Monoclonal antibody	Proteintech	66065-2-Ig	1:400
VE-cadherin Polyclonal antibody	Proteintech	27956-1-AP	1:200

Supplementary Table 3   List of siRNA Target Sequences

Name	siRNA/shRNA Target Sequence
siRNA targeting sequence: TANGO6 #1	CGATGCACTTAGTATCTCA
siRNA targeting sequence: TANGO6 #2	CCGAGAACCTTTGATCCAT
siRNA targeting sequence: RPB2	GTAGTGGAGTATATTGATA
siRNA targeting sequence: COPA	GTGGAAAAATTCCGTTCCA
siRNA targeting sequence: KPNB1 #1	AAACGACTTTGGTCATCAT
siRNA targeting sequence: KPNB1 #2	CCAGAGCACATCCGATAGA
siRNA targeting sequence: NSF #1	GGATAGGAATCAAGAAGTT
siRNA targeting sequence: NSF #2	GATCCTGAATACCGTGTGA
siRNA targeting sequence: NSF #3	GGAACGCACCACAATTGCA
siRNA targeting sequence: Arf1	GCCTGATCTTCGTGGTGGA
siRNA targeting sequence: COPB	GCAGAATTGCTAGAACCTTT
siRNA targeting sequence: TANGO1 #1	GCAATAACCTCAACTCTAT
siRNA targeting sequence: TANGO1 #2	ATAGAGTTGAGGTTATTGC
siRNA targeting sequence: LRRC59 #1	ACAAGGTGTTACAGCACATGA
siRNA targeting sequence: LRRC59 #2	TCATGTGCTGTAACACCTTGT
siRNA targeting sequence: AP1M1 #1	ACAACTTTGTTATCATCTA
siRNA targeting sequence: AP1M1 #2	TAGATGATAACAAAGTTGT
siRNA targeting sequence: TANGO2 #1	GGATGTGCTCAACAATGAA
siRNA targeting sequence: TANGO2 #2	CCAACAGGGATGAATTCTA
siRNA targeting sequence: TANGO2 #3	GAGACCAGAACCTATGAGT
siRNA targeting sequence: TANGO4 #1	GATACTAAGATCCAGAGAA
siRNA targeting sequence: TANGO4 #2	CCTACAGCGATGAATTCAA
siRNA targeting sequence: TANGO4 #3	CTCGAATACAATAAGGTTA
shRNA targeting sequence: mouse-Polr2b #1	GCCTGGTGTTACTAAGGAGAA
shRNA targeting sequence: mouse-Polr2b #2	GCGTCGTCTAAACTCTCCTAT
shRNA targeting sequence: mouse-Polr2b #3	CCAGCAGGAATCAACTCAATT
shRNA targeting sequence: mouse-Polr2b #4	GCGCATCGTGGCAACGTTACC

Supplementary Table 4 The list of cell lines and organisms in this study

Experimental models: cell lines / organisms	Source	IDENTIFIER
HeLa	Type Culture Collection of the Chinese Academy of Sciences, Shanghai, China	N/A
293T	Type Culture Collection of the Chinese Academy of Sciences, Shanghai, China	N/A
U251	Type Culture Collection of the Chinese Academy of Sciences, Shanghai, China	N/A
TANGO6 KO (U251)	This paper	N/A
TANGO6 +/- (HeLa)	This paper	N/A
Mouse: Tango6 +/- (conventional knock-out mice heterozygote)	Cyagen Biosciences	N/A
Mouse: Tango6 flox/flox	Cyagen Biosciences	N/A
Mouse: Rosa26Tango6/WT	Cyagen Biosciences	N/A
Mouse: Vav-iCre	Third Military Medical University, China	N/A
