# Supplementary material for: TANGO6 regulates cell proliferation via COPI vesicle-mediated RPB2 nuclear entry
Source: Nat Commun. 2024 Mar 15;15:2371. doi: 10.1038/s41467-024-46720-y (PMC10943085; doi:10.1038/s41467-024-46720-y)
Supplement: Supplementary file 3 — Reporting Summary [file 41467_2024_46720_MOESM3_ESM.pdf]

nature portfolio

Corresponding author(s): Li Li; Lingfei Luo

Last updated by author(s): Feb 7, 2024

Reporting Summary

Nature Portfolio wishes to improve the reproducibility of the work that we publish. This form provides structure for consistency and transparency in reporting. For further information on Nature Portfolio policies, see our Editorial Policies and the Editorial Policy Checklist.

Statistics

For all statistical analyses, confirm that the following items are present in the figure legend, table legend, main text, or Methods section.

n/a	Confirmed	
☐	☒	The exact sample size (*n*) for each experimental group/condition, given as a discrete number and unit of measurement
☐	☒	A statement on whether measurements were taken from distinct samples or whether the same sample was measured repeatedly
☐	☒	The statistical test(s) used AND whether they are one- or two-sided<br>*Only common tests should be described solely by name; describe more complex techniques in the Methods section.*
☒	☐	A description of all covariates tested
☒	☐	A description of any assumptions or corrections, such as tests of normality and adjustment for multiple comparisons
☐	☒	A full description of the statistical parameters including central tendency (e.g. means) or other basic estimates (e.g. regression coefficient) AND variation (e.g. standard deviation) or associated estimates of uncertainty (e.g. confidence intervals)
☐	☒	For null hypothesis testing, the test statistic (e.g. *F*, *t*, *r*) with confidence intervals, effect sizes, degrees of freedom and *P* value noted<br>*Give P values as exact values whenever suitable.*
☒	☐	For Bayesian analysis, information on the choice of priors and Markov chain Monte Carlo settings
☒	☐	For hierarchical and complex designs, identification of the appropriate level for tests and full reporting of outcomes
☒	☐	Estimates of effect sizes (e.g. Cohen's *d*, Pearson's *r*), indicating how they were calculated

Our web collection on statistics for biologists contains articles on many of the points above.

Software and code

Policy information about availability of computer code

Data collection	Zeiss Imaging Software (2.6) and Zeiss LSM700 and LSM880 was used for confocal image acquisition.<br>Beckman Coulter Moflo XDP and full spectrum flow cytometry (Cytek® Northern Lights) cell sorters were used for flow cytometry.<br>Roche LightCycler 96 Real-Time PCR system was used for Real-Time PCR analysis.
Data analysis	GraphPad Prism (v7.0e),Imaris (9.0.1) Zeiss Imaging Software (2.6), FlowJo (v10.5.3), Huygens Professional (Scientific Volume Imaging), Light Cycler 96 SW 1.1 Real-Time PCR Analysis, R (4.0), Image J (v1.53e), BioRender (https://biorender.com).

For manuscripts utilizing custom algorithms or software that are central to the research but not yet described in published literature, software must be made available to editors and reviewers. We strongly encourage code deposition in a community repository (e.g. GitHub). See the Nature Portfolio guidelines for submitting code & software for further information.

Data

Policy information about availability of data

All manuscripts must include a data availability statement. This statement should provide the following information, where applicable:

- Accession codes, unique identifiers, or web links for publicly available datasets
- A description of any restrictions on data availability
- For clinical datasets or third party data, please ensure that the statement adheres to our policy

The RNA-seq data had been deposited in the Sequence Read Archive under accession code PRJNA1065112. The mass spectrometry proteomics data have been

deposited to the ProteomeXchange Consortium via the iProX partner repository with the dataset identifier PXD049158.

Research involving human participants, their data, or biological material

Policy information about studies with human participants or human data. See also policy information about sex, gender (identity/presentation), and sexual orientation and race, ethnicity and racism.

Reporting on sex and gender	No reporting on sex and gender involved in this study.
Reporting on race, ethnicity, or other socially relevant groupings	No reporting on race, ethnicity, or other socially relevant groupings involved in this study.
Population characteristics	No population characteristics involved in this study.
Recruitment	No recruitment involved in this study.
Ethics oversight	The umbilical cord blood collection has been approved by the Ethics Committee of the Institute of Chongqing Medical University (Chongqing, China). The mice maintenance and experiment procedures of mice were complied with the guidelines approved by the Institutional Review Board of Chongqing Institute of Green and Intelligent Technology, Chinese Academy of Sciences(Chongqing, China).

Note that full information on the approval of the study protocol must also be provided in the manuscript.

Field-specific reporting

Please select the one below that is the best fit for your research. If you are not sure, read the appropriate sections before making your selection.

☒ Life sciences ☐ Behavioural & social sciences ☐ Ecological, evolutionary & environmental sciences

For a reference copy of the document with all sections, see nature.com/documents/nr-reporting-summary-flat.pdf

Life sciences study design

All studies must disclose on these points even when the disclosure is negative.

Sample size	It is generally considered that a sample size containing at least 3 biological replicates can provide adequate statistical power in biochemical analysis. We have described the exact sample size for each experiment in the figure legend of our manuscript.
Data exclusions	No data captured was excluded from the subsequent analyses.
Replication	The exact number of replication for all experiments was described in figure legends and our attempts at replication were successful.
Randomization	Samples and animals were allocated randomly.
Blinding	The experimenters were blinded to the animal genotype, grouping information and data analysis.

Behavioural & social sciences study design

All studies must disclose on these points even when the disclosure is negative.

Study description	*Briefly describe the study type including whether data are quantitative, qualitative, or mixed-methods (e.g. qualitative cross-sectional, quantitative experimental, mixed-methods case study).*
Research sample	*State the research sample (e.g. Harvard university undergraduates, villagers in rural India) and provide relevant demographic information (e.g. age, sex) and indicate whether the sample is representative. Provide a rationale for the study sample chosen. For studies involving existing datasets, please describe the dataset and source.*
Sampling strategy	*Describe the sampling procedure (e.g. random, snowball, stratified, convenience). Describe the statistical methods that were used to predetermine sample size OR if no sample-size calculation was performed, describe how sample sizes were chosen and provide a rationale for why these sample sizes are sufficient. For qualitative data, please indicate whether data saturation was considered, and what criteria were used to decide that no further sampling was needed.*
Data collection	*Provide details about the data collection procedure, including the instruments or devices used to record the data (e.g. pen and paper, computer, eye tracker, video or audio equipment) whether anyone was present besides the participant(s) and the researcher, and whether the researcher was blind to experimental condition and/or the study hypothesis during data collection.*

Timing	*Indicate the start and stop dates of data collection. If there is a gap between collection periods, state the dates for each sample cohort.*
Data exclusions	*If no data were excluded from the analyses, state so OR if data were excluded, provide the exact number of exclusions and the rationale behind them, indicating whether exclusion criteria were pre-established.*
Non-participation	*State how many participants dropped out/declined participation and the reason(s) given OR provide response rate OR state that no participants dropped out/declined participation.*
Randomization	*If participants were not allocated into experimental groups, state so OR describe how participants were allocated to groups, and if allocation was not random, describe how covariates were controlled.*

Ecological, evolutionary & environmental sciences study design

All studies must disclose on these points even when the disclosure is negative.

Study description	*Briefly describe the study. For quantitative data include treatment factors and interactions, design structure (e.g. factorial, nested, hierarchical), nature and number of experimental units and replicates.*
Research sample	*Describe the research sample (e.g. a group of tagged Passer domesticus, all Stenocereus thurberi within Organ Pipe Cactus National Monument), and provide a rationale for the sample choice. When relevant, describe the organism taxa, source, sex, age range and any manipulations. State what population the sample is meant to represent when applicable. For studies involving existing datasets, describe the data and its source.*
Sampling strategy	*Note the sampling procedure. Describe the statistical methods that were used to predetermine sample size OR if no sample-size calculation was performed, describe how sample sizes were chosen and provide a rationale for why these sample sizes are sufficient.*
Data collection	*Describe the data collection procedure, including who recorded the data and how.*
Timing and spatial scale	*Indicate the start and stop dates of data collection, noting the frequency and periodicity of sampling and providing a rationale for these choices. If there is a gap between collection periods, state the dates for each sample cohort. Specify the spatial scale from which the data are taken*
Data exclusions	*If no data were excluded from the analyses, state so OR if data were excluded, describe the exclusions and the rationale behind them, indicating whether exclusion criteria were pre-established.*
Reproducibility	*Describe the measures taken to verify the reproducibility of experimental findings. For each experiment, note whether any attempts to repeat the experiment failed OR state that all attempts to repeat the experiment were successful.*
Randomization	*Describe how samples/organisms/participants were allocated into groups. If allocation was not random, describe how covariates were controlled. If this is not relevant to your study, explain why.*
Blinding	*Describe the extent of blinding used during data acquisition and analysis. If blinding was not possible, describe why OR explain why blinding was not relevant to your study.*

Did the study involve field work? ☐ Yes ☐ No

Field work, collection and transport

Field conditions	*Describe the study conditions for field work, providing relevant parameters (e.g. temperature, rainfall).*
Location	*State the location of the sampling or experiment, providing relevant parameters (e.g. latitude and longitude, elevation, water depth).*
Access & import/export	*Describe the efforts you have made to access habitats and to collect and import/export your samples in a responsible manner and in compliance with local, national and international laws, noting any permits that were obtained (give the name of the issuing authority, the date of issue, and any identifying information).*
Disturbance	*Describe any disturbance caused by the study and how it was minimized.*

Reporting for specific materials, systems and methods

We require information from authors about some types of materials, experimental systems and methods used in many studies. Here, indicate whether each material, system or method listed is relevant to your study. If you are not sure if a list item applies to your research, read the appropriate section before selecting a response.

Materials & experimental systems

n/a	Involved in the study
	[x] Antibodies
	[x] Eukaryotic cell lines
[x]	Palaeontology and archaeology
	[x] Animals and other organisms
[x]	Clinical data
[x]	Dual use research of concern
[x]	Plants

Methods

n/a	Involved in the study
[x]	ChIP-seq
	[x] Flow cytometry
[x]	MRI-based neuroimaging

Antibodies

Antibodies used

The following antibodies were used for western blot (WB) analysis or immunofluorescent staining (IF) are listed in supplementary table 2: Goat polyclonal anti-GFP (Abcam, #ab6658, IF: 1:400, WB: 1:2000), Goat polyclonal anti-Calnexin (Abcam, #ab219644, IF: 1:400, WB: 1:1000), Mouse monoclonal anti-beta-Tubulin (Invitrogen, #MA5-11732, WB: 1:1000), Rabbit polyclonal anti-TMCO7 (TANGO6) (Invitrogen, #PA5-59510, IF: 1:400, WB: 1:1000), Mouse monoclonal anti-Lamin B1 (Proteintech, #66095-1-1g, IF: 1:400), Mouse monoclonal anti-PCNA (Proteintech, #60097-1-1g, IF: 1:400, WB: 1:1000), Mouse monoclonal anti-GAPDH (Proteintech, #60004-1-Ig, WB: 1:2000), Rabbit Recombinant monoclonal anti-Histone 3 (Bimake, #A5885, WB: 1:2000), Mouse Recombinant monoclonal anti-HA (Bimake, #A5969, WB: 1:1000), Rabbit Recombinant monoclonal anti-TGN46 (Bimake, #A5460, IF: 1:400, WB: 1:1000), Rabbit Recombinant monoclonal anti-Calreticulin (Bimake, #A5231, WB: 1:1000), Mouse Monoclonal anti-FLAG (Sigma-Aldrich, #F1804, WB: 1:1000), Mouse monoclonal anti-COPA (Santa Cruz, #SC-398099, IF: 1:200, WB: 1:500), Mouse monoclonal anti-COPB (Santa Cruz, #SC-393615, IF: 1:200, WB: 1:500), Mouse monoclonal anti-COPD (Santa Cruz, #SC-515549, IF: 1:200, WB: 1:500), Mouse monoclonal anti-COPE (Santa Cruz, # SC-133194, IF: 1:200, WB: 1:500), Mouse monoclonal anti-COPG (Santa Cruz, #SC-393977, IF: 1:200, WB: 1:500), Mouse monoclonal anti-COPZI (Santa Cruz, #SC-398081, IF: 1:200, WB: 1:500), Mouse monoclonal anti-KPNB1 (Santa Cruz, #SC-137016, IF: 1:200, WB: 1:500), Mouse monoclonal: anti-ERGIC53(F-3) (Santa Cruz, #SC-398777, IF: 1:200, WB: 1:500), Mouse monoclonal anti-ERGIC53(B-4) (Santa Cruz, #SC-398893, IF: 1:200, WB: 1:500), Mouse monoclonal anti-ERGIC53(C-6) (Santa Cruz, #SC-365158, IF: 1:200, WB: 1:500), Mouse monoclonal anti-Sec31A (Santa Cruz, #SC-376587, IF: 1:200, WB: 1:500), Mouse monoclonal anti-Calnexin (Santa Cruz, #SC-70481, IF: 1:100, WB: 1:400), Rabbit polyclonal anti-PH3 (Santa Cruz, #SC-8656, IF: 1:200, WB: 1:400), Mouse monoclonal anti-POLR2A (RPB1) (Santa Cruz, #SC-55492, IF: 1:200, WB: 1:1000), Mouse monoclonal anti-POLR2B (RPB2) (Santa Cruz, #SC-166803, IF: 1:200, WB: 1:500), Mouse monoclonal anti-CDK1 (Santa Cruz, #SC-53219, IF: 1:200, WB: 1:1000), Rabbit monoclonal anti-GM130 (Cell Signaling Technology, #124805, IF: 1:400, WB: 1:1000), Rabbit monoclonal Ki-67(D3B5) (Cell Signaling, #Technology 9129T, IF: 1:400, WB: 1:1000), Mouse monoclonal anti-GM130 (BD Bioscience, #610822, IF: 1:400, WB: 1:1000), BD PharmingenTM Purified Rabbit Anti-Active Caspase-3 (BD Bioscience, #559565, IF: 1:400, WB: 1:1000), Rabbit Polyclonal anti-Emerin (Proteintech, #10351-1-AP, IF: 1:400, WB: 1:1000), Mouse monoclonal anti-DsRed (Santa Cruz, #SC-390909, IF: 1:200, WB: 1:500), Mouse monoclonal anti-Thymidine kinase 1 (Santa Cruz, #SC-377211, IF: 1:200, WB: 1:500), Mouse monoclonal anti-Cyclin E (Santa Cruz, #SC-377100, IF: 1:200, WB: 1:500), Mouse monoclonal anti-Cyclin A (Santa Cruz, #SC-271682, IF:1:200, WB: 1:500), Goat anti-Mouse IgG (H+L) Secondary Antibody, HRP (Invitrogen, #31430, WB: 1:2000), Goat anti-Rabbit IgG (H+L) Secondary Antibody,HRP (Invitrogen, #32260, WB: 1:2000), Alexa Fluor 488-Donkey anti-Goat IgG (H+L) (Invitrogen, #A32814, IF: 1:400), Alexa Fluor 555-Donkey anti-Mouse IgG (H+L) (Invitrogen, #A31570R, IF: 1:400), Alexa Fluor 647-Donkey anti-Rabbit IgG (H+L) (Invitrogen, #A31573, IF: 1:400), FITC anti-mouse Lineage Cocktail with Isotype Ctrl (Biolegend, #133301, 2µl/105 cells), Rabbit monoclonal PE/Cyanine anti-mouse Ly-6A/E (Sca-1) (Biolegend, #108113, 0.2µl/105 cells), Rabbit monoclonal APC anti-mouse CD117 (c-Kit) (Biolegend, #105811, 0.5µl/105 cells), Rabbit monoclonal APC/Cyanine7 anti-mouse TER-119/Erythroid (Biolegend, #116223, 1.25µl/105 cells), Rabbit monoclonal APC anti-mouse Ly-6G/Ly-6C(Gr-1) (Biolegend, #108411, 1.25µl/105 cells), Rabbit monoclonal FITC anti-mouse/human CD11b (Biolegend, #101205, 0.5µl/105 cells), Alexa Fluor 700 anti-mouse Ki-67 Antibody (Biolegend, #652419, 0.03µl/105 cells), Rabbit anti-Goat IgG (H+L) Secondary Antibody, HRP (Pierce, #31402, WB: 1:2000), abberior STAR RED abberior STRED(IF: 1:200), abberior STAR 580 abberior ST580(IF: 1:200), Rabbit Polyclonal anti-Giantin (ABclonal, #A22233, IF: 1:200), Rabbit monoclonal anti-VE Cadherin (ABclonal, #A22659, IF: 1:200), Rabbit monoclonal anti-PECAM1 (ABclonal, #A19014, IF: 1:200), PE anti-mouse CD150 (SLAM) antibody (Biolegend, #162605, 0.125µl/105 cells), APC/Cyanine7 anti-mouse CD48 antibody (Biolegend, #103431, 0.125µl/105 cells), CLTC Monoclonal antibody (Proteintech, #66487-1-Ig, IF: 1:400), POLR2I Polyclonal antibody (Proteintech, #17270-1-AP, IF: 1:400), Mouse monoclonal anti-POLR2E (Santa Cruz, #sc-390979, IF: 1:200, WB: 1:500), POLR2F Polyclonal antibody (Proteintech, #15334-1-AP, IF: 1:200), POLR2C Polyclonal antibody (Proteintech, #13428-1-AP, IF: 1:400), POLR2H Polyclonal antibody (Proteintech, #15086-1-AP, IF: 1:400), POLR2J Polyclonal antibody (Proteintech, #16403-1-AP, IF: 1:400), POLR2D Polyclonal antibody (Proteintech, #16093-1-AP, IF: 1:400), POLR2L Polyclonal antibody (Proteintech, #15779-1-AP, IF: 1:400), Anti-POLR2G Polyclonal antibody (Solarbio, #K004815P, IF: 1:200), Rabbit Polyclonal anti-POLR2K (GeneTex, #GTX132871, IF: 1:400), GS28 Polyclonal antibody (Proteintech, #16106-1-AP, IF: 1:400), golgin97 Polyclonal antibody (Proteintech, #12640-1-AP, IF: 1:400), LRRC59 Polyclonal antibody (Proteintech, #27208-1-AP, IF: 1:400), CD31 Monoclonal antibody (Proteintech, #66065-2-Ig, IF: 1:400), VE-cadherin Polyclonal antibody (Proteintech, #27956-1-AP, IF: 1:200), EGF polyclonal antibody (Proteintech, #27141-1-AP, WB: 1:500) and Collagen Type I Polyclonal antibody (Proteintech, 14695-1-AP, WB: 1:500), Albumin Monoclonal antibody (Proteintech, 66051-1-Ig, IF: 1:200).

Validation

All antibodies were commercial in origin and validated by the company. Information regarding validation processes can be easily accessed on the company's web site using the product numbers listed above. These company's web site show that these antibodies except for rabbit anti-TMCO7(TANGO6) are suitable for mammals, including mice and human cell line. The verification result about a human antibody, rabbit anti-TMCO7(TANGO6), against mouse TANGO6 antigen has been verified in Extended Data Fig. 8f.

Eukaryotic cell lines

Policy information about cell lines and Sex and Gender in Research

Cell line source(s)	293T, HeLa and U251 cells were ordered from Type Culture Collection of the Chinese Academy of Sciences, Shanghai, China
Authentication	All the cell lines were confirmed by STR identification and the morphologies were consistent with previous reports.
Mycoplasma contamination	The mycoplasma of all the cell lines were detected by mycoplasma PCR detection kit (Beyotime), and mycoplasma inhibitor (Beyotime) was added in medium during cell culture.
Commonly misidentified lines (See ICLAC register)	No commonly misidentified lines were used in this study.

Palaeontology and Archaeology

Specimen provenance	*Provide provenance information for specimens and describe permits that were obtained for the work (including the name of the issuing authority, the date of issue, and any identifying information). Permits should encompass collection and, where applicable, export.*
Specimen deposition	*Indicate where the specimens have been deposited to permit free access by other researchers.*
Dating methods	*If new dates are provided, describe how they were obtained (e.g. collection, storage, sample pretreatment and measurement), where they were obtained (i.e. lab name), the calibration program and the protocol for quality assurance OR state that no new dates are provided.*

☐ Tick this box to confirm that the raw and calibrated dates are available in the paper or in Supplementary Information.

Ethics oversight	*Identify the organization(s) that approved or provided guidance on the study protocol, OR state that no ethical approval or guidance was required and explain why not.*

Note that full information on the approval of the study protocol must also be provided in the manuscript.

Animals and other research organisms

Policy information about studies involving animals; ARRIVE guidelines recommended for reporting animal research, and Sex and Gender in Research

Laboratory animals	Mice were bred at 23°C, with 40-60% relative humidity, 12hrs light cycle (8am-8pm).<br>According to the purpose of each experiment, WT C57BL/6J (The Jackson Laboratory, #000664), Tango6 +/- (conventional knock-out mice heterozygote, Cyagen Biosciences) , Tango6 flox/flox (Cyagen Biosciences), Rosa26Tango6/WT (Cyagen Biosciences) and Vav-iCre (Third Military Medical University, China).<br>Mice were used and both genders were used in the entire study. E9.5, E12.5, E14.5, E15.5 and E16.5 mice embryos were used.
Wild animals	No wild animals involved in this study.
Reporting on sex	In this study, we focused on mice early embryonic hematopoietic development, and E9.5-E15.5 mice embryos were used. No adult mice were involved in this study, therefore, we did not need to consider sex difference in experiment design.
Field-collected samples	The study did not involve samples collected from the field.
Ethics oversight	All animal experiments were approved by the Institutional Review Board of Chongqing Institute of Green and Intelligent Technology, Chinese Academy of Sciences(Chongqing, China)..

Note that full information on the approval of the study protocol must also be provided in the manuscript.

Clinical data

Policy information about clinical studies

All manuscripts should comply with the ICMJEguidelines for publication of clinical research and a completedCONSORT checklist must be included with all submissions.

Clinical trial registration	*Provide the trial registration number from ClinicalTrials.gov or an equivalent agency.*
Study protocol	*Note where the full trial protocol can be accessed OR if not available, explain why.*
Data collection	*Describe the settings and locales of data collection, noting the time periods of recruitment and data collection.*
Outcomes	*Describe how you pre-defined primary and secondary outcome measures and how you assessed these measures.*

Dual use research of concern

Policy information about [dual use research of concern](#)

Hazards

Could the accidental, deliberate or reckless misuse of agents or technologies generated in the work, or the application of information presented in the manuscript, pose a threat to:

No	Yes	
☐	☐	Public health
☐	☐	National security
☐	☐	Crops and/or livestock
☐	☐	Ecosystems
☐	☐	Any other significant area

Experiments of concern

Does the work involve any of these experiments of concern:

No	Yes	
☐	☐	Demonstrate how to render a vaccine ineffective
☐	☐	Confer resistance to therapeutically useful antibiotics or antiviral agents
☐	☐	Enhance the virulence of a pathogen or render a nonpathogen virulent
☐	☐	Increase transmissibility of a pathogen
☐	☐	Alter the host range of a pathogen
☐	☐	Enable evasion of diagnostic/detection modalities
☐	☐	Enable the weaponization of a biological agent or toxin
☐	☐	Any other potentially harmful combination of experiments and agents

Plants

Seed stocks	*Report on the source of all seed stocks or other plant material used. If applicable, state the seed stock centre and catalogue number. If plant specimens were collected from the field, describe the collection location, date and sampling procedures.*
Novel plant genotypes	*Describe the methods by which all novel plant genotypes were produced. This includes those generated by transgenic approaches, gene editing, chemical/radiation-based mutagenesis and hybridization. For transgenic lines, describe the transformation method, the number of independent lines analyzed and the generation upon which experiments were performed. For gene-edited lines, describe the editor used, the endogenous sequence targeted for editing, the targeting guide RNA sequence (if applicable) and how the editor was applied.*
Authentication	*Describe any authentication procedures for each seed stock used or novel genotype generated. Describe any experiments used to assess the effect of a mutation and, where applicable, how potential secondary effects (e.g. second site T-DNA insertions, mosiacism, off-target gene editing) were examined.*

ChIP-seq

Data deposition

☐ Confirm that both raw and final processed data have been deposited in a public database such as [GEO](#).

☐ Confirm that you have deposited or provided access to graph files (e.g. BED files) for the called peaks.

Data access links<br>*May remain private before publication.*	*For "Initial submission" or "Revised version" documents, provide reviewer access links. For your "Final submission" document, provide a link to the deposited data.*
Files in database submission	*Provide a list of all files available in the database submission.*
Genome browser session<br>(e.g. [UCSC](#))	*Provide a link to an anonymized genome browser session for "Initial submission" and "Revised version" documents only, to enable peer review. Write "no longer applicable" for "Final submission" documents.*

Methodology

Replicates	*Describe the experimental replicates, specifying number, type and replicate agreement.*
Sequencing depth	*Describe the sequencing depth for each experiment, providing the total number of reads, uniquely mapped reads, length of reads and whether they were paired- or single-end.*
Antibodies	*Describe the antibodies used for the ChIP-seq experiments; as applicable, provide supplier name, catalog number, clone name, and lot number.*
Peak calling parameters	*Specify the command line program and parameters used for read mapping and peak calling, including the ChIP, control and index files used.*
Data quality	*Describe the methods used to ensure data quality in full detail, including how many peaks are at FDR 5% and above 5-fold enrichment.*
Software	*Describe the software used to collect and analyze the ChIP-seq data. For custom code that has been deposited into a community repository, provide accession details.*

Flow Cytometry

Plots

Confirm that:

- [x] The axis labels state the marker and fluorochrome used (e.g. CD4-FITC).
- [x] The axis scales are clearly visible. Include numbers along axes only for bottom left plot of group (a 'group' is an analysis of identical markers).
- [x] All plots are contour plots with outliers or pseudocolor plots.
- [x] A numerical value for number of cells or percentage (with statistics) is provided.

Methodology

Sample preparation	Mice HSPCs isolation: Whole fetal liver were cut into pieces and then digested with collagenase IV (Sangon Biotech) at 37°C for 30-60 min. Single-cell suspensions were obtained by filtration of 70 µm cell strainer. Erythrocytes were lysed using red blood cell lysis buffer (Beyotime) and then incubated with the corresponding antibodies in the dark at 4°C for 1 h, and the sediment was resuspended by 5% FBS (in 1 X PBS) for FACS analysis and RNA extraction by REPLI-g WTA Single Cell Kit (QIAGEN).
Instrument	Beckman Coulter Moflo XDP cell sorter
Software	FlowJo Software (10.5.3) of Beckman Coulter Moflo XDP cell sorter was used for cell sorting
Cell population abundance	About 3x10^4 LSK (Lin-Sca-1+c-Kit+) cells in mice fetal livers were collected by Moflo XDP cell sorter with 99% of purity to do FACS analysis (including LSK/LK fractions and LT/ST fractions). About 300 LSK cells in mice fetal livers were collected by Moflo XDP cell sorter with 99% of purity to do RNA extraction by REPLI-g WTA Single Cell Kit (QIAGEN).
Gating strategy	Gating strategy for LSK cells isolated from E13.5, E15.5 and E16.5 mice fetal livers (WT and cKO) samples as presented in Fig. 6f. Samples of mice LT/ST-HSC cells isolated from E15.5 mice fetal livers (WT and cKO) samples as presented in Supplementary Fig. 8m, accordingly to specific gene markers (CD150hi/CD48lo and CD150lo/CD48lo).

- [x] Tick this box to confirm that a figure exemplifying the gating strategy is provided in the Supplementary Information.

Magnetic resonance imaging

Experimental design

Design type	*Indicate task or resting state; event-related or block design.*
Design specifications	*Specify the number of blocks, trials or experimental units per session and/or subject, and specify the length of each trial or block (if trials are blocked) and interval between trials.*
Behavioral performance measures	*State number and/or type of variables recorded (e.g. correct button press, response time) and what statistics were used to establish that the subjects were performing the task as expected (e.g. mean, range, and/or standard deviation across subjects).*

Acquisition

Imaging type(s) | Specify: functional, structural, diffusion, perfusion.

Field strength | Specify in Tesla

Sequence & imaging parameters | Specify the pulse sequence type (gradient echo, spin echo, etc.), imaging type (EPI, spiral, etc.), field of view, matrix size, slice thickness, orientation and TE/TR/flip angle.

Area of acquisition | State whether a whole brain scan was used OR define the area of acquisition, describing how the region was determined.

Diffusion MRI ☐ Used ☐ Not used

Preprocessing

Preprocessing software | Provide detail on software version and revision number and on specific parameters (model/functions, brain extraction, segmentation, smoothing kernel size, etc.).

Normalization | If data were normalized/standardized, describe the approach(es): specify linear or non-linear and define image types used for transformation OR indicate that data were not normalized and explain rationale for lack of normalization.

Normalization template | Describe the template used for normalization/transformation, specifying subject space or group standardized space (e.g. original Talairach, MNI305, ICBM152) OR indicate that the data were not normalized.

Noise and artifact removal | Describe your procedure(s) for artifact and structured noise removal, specifying motion parameters, tissue signals and physiological signals (heart rate, respiration).

Volume censoring | Define your software and/or method and criteria for volume censoring, and state the extent of such censoring.

Statistical modeling & inference

Model type and settings | Specify type (mass univariate, multivariate, RSA, predictive, etc.) and describe essential details of the model at the first and second levels (e.g. fixed, random or mixed effects; drift or auto-correlation).

Effect(s) tested | Define precise effect in terms of the task or stimulus conditions instead of psychological concepts and indicate whether ANOVA or factorial designs were used.

Specify type of analysis: ☐ Whole brain ☐ ROI-based ☐ Both

Statistic type for inference | Specify voxel-wise or cluster-wise and report all relevant parameters for cluster-wise methods.

(See Eklund et al. 2016)

Correction | Describe the type of correction and how it is obtained for multiple comparisons (e.g. FWE, FDR, permutation or Monte Carlo).

Models & analysis

n/a	Involved in the study
☐	☐ Functional and/or effective connectivity
☐	☐ Graph analysis
☐	☐ Multivariate modeling or predictive analysis

Functional and/or effective connectivity | Report the measures of dependence used and the model details (e.g. Pearson correlation, partial correlation, mutual information).

Graph analysis | Report the dependent variable and connectivity measure, specifying weighted graph or binarized graph, subject- or group-level, and the global and/or node summaries used (e.g. clustering coefficient, efficiency, etc.).

Multivariate modeling and predictive analysis | Specify independent variables, features extraction and dimension reduction, model, training and evaluation metrics.
