# Supplementary material for: TANGO6 regulates cell proliferation via COPI vesicle-mediated RPB2 nuclear entry
Source: Nat Commun. 2024 Mar 15;15:2371. doi: 10.1038/s41467-024-46720-y (PMC10943085; doi:10.1038/s41467-024-46720-y)
Supplement: Supplementary file 4 — Description of Additional Supplementary Files [file 41467_2024_46720_MOESM4_ESM.pdf]

Description of Additional Supplementary Files

Supplementary Data 1. Mass spectrum results. TANGO6 or FLAG cross linked beads are used to pull down endogenous/ exogenous proteins. These beads are sent to SIMM (Shanghai Institute of Materia Medica, Chinese Academy of Science) to perform mass spectrometry (Orbitrap fusion). The Maxquant (1.6.2.3) software package is used to analyze the data from mass spectra. Peptide identification is filtered at a false discovery rate (FDR) < 1%. The candidate proteins are identified from high to low scores. TANGO6 is used as a positive control.

Supplementary Data 2. Pulse-chase assay.
The stable isotope [2,3-13C2]alanine is used to label proteins and followed their secretion. The cell pellet and medium supernatant are collected at different time points (0 h, 2 h and 6 h). These samples are sent to Metabo-Profile Biotechnology (Shanghai) Co., Ltd to detect relative abundance of [2,3-13C2]alanine. The project is performed under the guidance of Quality Management System ISO 9001:2015 (QAIC/CN/170149).
